# Necrotizing Mediastinitis Following Dental Extraction: A Case Report

**DOI:** 10.5811/cpcem.2021.11.54567

**Published:** 2022-01-28

**Authors:** Justin Pinkston, Charles A. Khoury, Jaron D. Raper

**Affiliations:** University of Alabama at Birmingham, Department of Emergency Medicine, Birmingham, Alabama

**Keywords:** descending necrotizing mediastinitis, odontogenic infections, facial abscess, septic shock, pericarditis, dental emergency, case report

## Abstract

**Introduction:**

Necrotizing mediastinitis is a rare complication following a dental procedure. It is frequently lethal and requires prompt diagnosis with advanced imaging, administration of broad-spectrum antimicrobials, and early surgical consultation.

**Case Report:**

A 19-year-old, otherwise healthy male presented to the emergency department with chest pain, muffled voice, and facial and neck swelling six days following dental extraction. He was found to have a retropharyngeal abscess causing necrotizing mediastinitis and septic shock. The patient was started on broad-spectrum antimicrobials, required 10 surgical procedures, and experienced a prolonged hospitalization.

**Conclusion:**

Consider necrotizing mediastinitis in patients presenting with chest pain and signs of retropharyngeal infection after dental procedures. Prompt imaging, antimicrobial therapy, and surgical consultation is critical in treating this frequently fatal disease.

## INTRODUCTION

Odontogenic infections are largely polymicrobial, and occasionally they may spread down the cervical fascial planes, resulting in complications including descending necrotizing mediastinitis, airway obstruction, and pericarditis.^1^ Treatment consists of prompt identification and drainage of the odontogenic infection source by a surgical subspecialty. However, the mortality rate remains high and is estimated to be between 25–40% and as high as 60% despite advancements in computed tomography (CT) imaging, directed antibiotic therapy, and improvements in intensive care and surgical drainage.^2^,^3^

## CASE REPORT

**Image 1:**
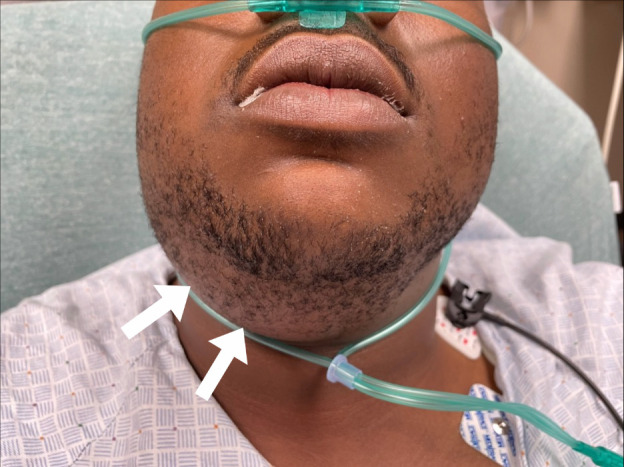
Photograph of patient’s lower jaw and neck demonstrating sublingual and submandibular swelling (arrows).

A 19-year-old, otherwise healthy male presented to the emergency department (ED) with worsening chest pain, muffled voice, facial, and neck swelling (Image 1) six days following a right-sided wisdom tooth extraction.

On presentation, the patient’s vital signs included a blood pressure of 117/77 millimeters of mercury (mm Hg), heart rate of 130 beats per minute, respiration rate of 28 breaths per minute, and an oral temperature of 98.4°F. Physical examination revealed mild respiratory distress and a muffled voice with diffuse submandibular swelling. The patient demonstrated trismus but was able to protrude his tongue, albeit painfully. The submandibular space on the right side was particularly tender, swollen, and fluctuant, without associated erythema. Crepitus was noted under the right side of the mandible and extended to the ipsilateral clavicle. Breath sounds were diminished on the right. Cardiac auscultation demonstrated a distant first and second heart sounds without appreciable murmur, rub, or gallop. During the examination, the patient reported orthopnea on several occasions, stating that he would prefer to remain upright in the stretcher.

**Image 2:**
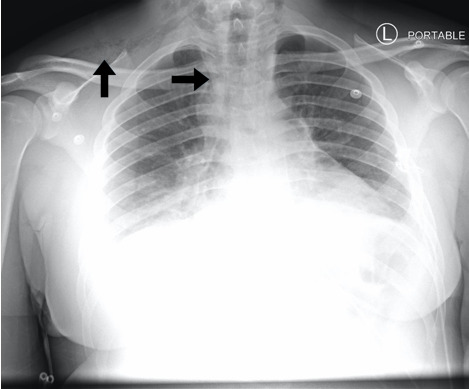
Chest radiograph demonstrating pneumomediastinum and subcutaneous emphysema just above the right clavicle (arrows).

A chest radiograph was obtained to evaluate the crepitus noted over the right clavicle (Image 2). The radiograph demonstrated pneumomediastinum and subcutaneous emphysema, which was concerning for esophageal perforation. As a result, a CT chest with contrast and esophagram were ordered. Initial labs included a white blood cell (WBC) count of 8.65 10^3/cells per cubic millimeter (reference range 4500 to 11,000 WBCs per microliter) and lactic acid of 3.3 millimoles per liter (mmol/L) (reference range 0.5–2.2 mmol/L).

**Image 3:**
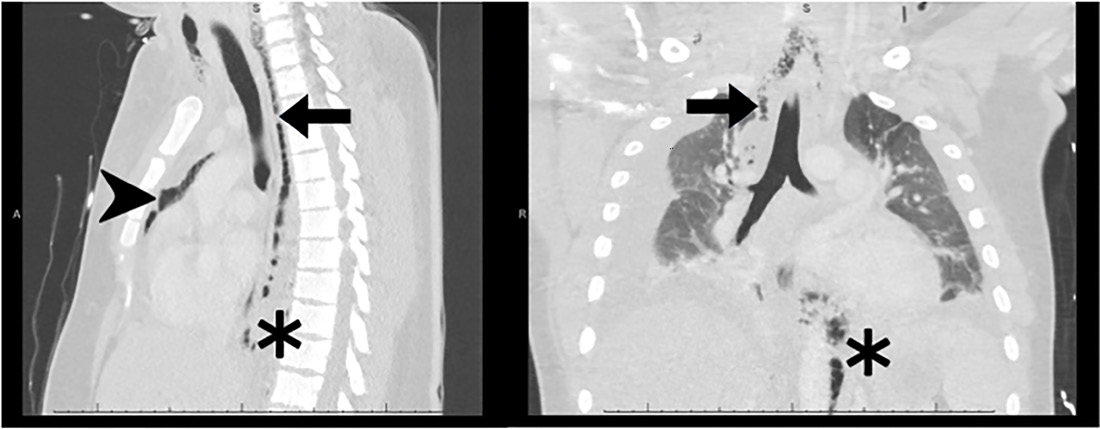
Computed tomography of the chest with intravenous contrast in coronal (right) and sagittal (left) planes, demonstrating mediastinal air and fluid tracking from the retropharyngeal space (arrows), extensive pneumo-mediastinum (arrowhead), and retrocrural subcutaneous gas (asterisks).

Computed tomography imaging demonstrated mediastinal air, small bilateral pleural effusions, and subcutaneous air tracking into the neck, all of which were concerning for esophageal perforation (Image 3). Following source identification by CT, the patient received vancomycin, piperacillin-tazobactam, clindamycin, metronidazole, and fluconazole to treat for necrotizing soft tissue infection (NSTI), severe sepsis, and esophageal perforation.

**CPC-EM Capsule**

What do we already know about this clinical entity?

*Odontogenic infections can rarely cause descending necrotizing infections of the mediastinum, with mortality ranging from 25–60%*.

What makes this presentation of disease reportable?

*This patient suffered a complicated and protracted course of necrotizing mediastinitis following an otherwise uncomplicated dental extraction*.

What is the major learning point?

*Consider necrotizing mediastinitis in patients with chest pain after dental procedures. Early imaging, antimicrobials, and surgical consultation is critical to management*.

How might this improve emergency medicine practice?

*Readers will have an improved ability to recognize and manage patients suffering from a life-threatening complication of dental extraction*.

Thoracic surgery and oral and maxillofacial surgery were emergently consulted. Following the surgeons’ bedside evaluation, the patient was taken to the operating room (OR). While in the ED, the patient remained normotensive and did not require a definitive airway. He was intubated in the OR using video-assisted laryngoscopy. Oral and maxillofacial surgery performed an incision and drainage of the patient’s facial abscess, and thoracic surgery performed a right-sided, video-assisted thoracoscopic surgery to drain the mediastinal abscess. Following surgery, the patient was admitted to the surgical intensive care unit (SICU) with septic shock and was placed on norepinephrine and phenylephrine due to persistent mean arterial pressure less than 60 mm Hg (reference range: normal greater than 65 mm Hg) and a peak lactic acid of 12 mmol/L.

The patient subsequently underwent numerous washouts over the following weeks and was weaned off vasopressors and mechanical ventilation after 35 days in the SICU. He was discharged home at 42 days. At his three-week follow-up appointment, he reported persistent oral pain, left-sided chin numbness, and limited opening of his mouth secondary to pain.

## DISCUSSION

Complications from odontogenic surgeries such as descending necrotizing mediastinitis, retropharyngeal abscess, and pericarditis are rare. Most cases are reported in dental and oral surgery literature, with relatively few in the emergency medicine literature. The incidence of complications from these surgeries ranges from 1–30%, with the most common complications being alveolar osteitis, postoperative hemorrhage, wound dehiscence, and fracture of the bone cortices. More serious infections including NSTI and complex abscesses have a reported rate of less than 2%, although some studies report rates as high as 15%. The variance in reported rate is most likely due to inconsistencies in defining NSTI, with the more aggressive pathology occurring in less than 2% of reported cases.^2^

As estimated 60–70% of all cases of descending necrotizing mediastinitis are secondary to odontogenic or cervicofacial infections. These necrotizing infections can carry a mortality rate as high as 60% and are frequently associated with pleural and pericardial effusions, sepsis, and multisystem organ failure.^3^ Patients presenting to the ED following odontogenic surgeries should be thoroughly evaluated for airway compromise, complicating infections, and sepsis.^5^

Airway compromise may present with subtle but specific findings including muffled voice secondary to retropharyngeal abscess. Changes in tongue articulation may be evident secondary to sublingual space infections.^5^ Patients with more advanced airway compromise may present with drooling, in a sniffing position, or with accessory muscle use.^5^ Endotracheal intubation of these patients may be challenging due to deviation of the airway and associated trismus. Emergency physicians should prepare for difficult airways in these patients by planning for nasopharyngeal fiberoptic intubation and potential cricothyrotomy.^5^

Once the airway is secure, the emergency physician should focus on identifying the source of infection and assessing for potential spread. Surgical exploration is the gold standard for the diagnosis of NSTI.^6^ Magnetic resonance imaging (MRI) is reported to attain a 100% sensitivity and 86% specificity for diagnosing NSTI.^6^ However, MRI is not always a feasible option in the ED due to timing and clinical instability. Computed tomography is reported to attain a 86% sensitivity and 92% specificity based on the presence of fascial air, muscle or fascial edema, fluid tracking, lymphadenopathy, and subcutaneous edema.^6^ If CT is unavailable, posteroanterior and lateral radiography of the neck and chest may demonstrate gas in soft tissue space, mediastinal widening, and increased thickness of the retropharyngeal tissues.^5^

Emergent surgical intervention is an important step in improving patient outcomes for those with NSTI.^2^ Primary treatment includes surgical drainage of the pharyngeal or odontogenic infection source.^2^ For NSTI, the Infectious Diseases Society of America recommends vancomycin or linezolid plus piperacillin-tazobactam or a carbapenem; if there is concern for esophageal perforation, antifungal coverage with fluconazole or micafungin should be added.^7^ Despite these therapies, the mortality rate remains as high as 60% for descending necrotizing mediastinitis.^5^

## CONCLUSION

Life-threatening odontogenic infections are rare complications of dental procedures. However, complications such as descending necrotizing mediastinitis and associated septic shock carry mortality rates as high as 60%.^5^ Clinicians should have a high index of suspicion for necrotizing soft tissue infections in patients presenting in respiratory distress following recent dental procedures. Prompt airway assessment and management, directed imaging, antimicrobial therapy, and surgical consultation are all essential for improving patient outcomes.
